# Loading IR820 Using Multifunctional Dendrimers with Enhanced Stability and Specificity

**DOI:** 10.3390/pharmaceutics10030077

**Published:** 2018-06-28

**Authors:** Hui Liu, Jingjing Wang

**Affiliations:** 1 Institute for Clean Energy and Advanced Materials, Faculty of Materials and Energy, Southwest University, Chongqing 400715, China; jingjingwang0502@gmail.com; 2 Chongqing Engineering Research Center for Micro-Nano Biomedical Materials and Devices, Chongqing 400715, China

**Keywords:** poly(amidoamine) dendrimers, new indocyanine green (IR820), stability, targeted delivery, cancer cells

## Abstract

Cyanine dyes are promising candidates in biomedical applications. Although various delivery systems have been developed to enhance their properties, their dendrimer-based delivery systems are seldom investigated. Herein, amine-terminated generation 5 poly(amidoamine) (G5.NH_2_) dendrimers and new indocyanine green (IR820) dyes were chosen as models to study the loading ability of dendrimers for cyanine dynes. G5.NH_2_ dendrimers were pre-modified with arginine-glycine-aspartic (RGD) peptides, poly(ethylene glycol) chains, and acetyl groups to be endowed with cancer cell specificity and biocompatibility. The formed Ac-PR dendrimers were used to load IR820, followed by thorough characterization. The loaded number of IR820 was estimated to be 6.7 per dendrimer. The stability of IR820 was improved through dendrimer loading, which was proved by their UV-vis spectra under different kinds of storage conditions. In addition, the formed Ac-PR dendrimers can retain the loaded IR820 effectively. Their cytocompatibility was desirable under the studied conditions. Their cellular uptake behaviors were demonstrated to be enhanced by RGD modification, showing concentration-, co-incubation time-, and α_v_β_3_ integrin receptor-dependent properties, displaying a cytoplasm-location. The findings from this work demonstrated the versatile loading and delivery capacity of dendrimers for near-infrared (NIR) dyes, providing fundamental data for the development of dendrimer/NIR dye systems for biomedical applications, especially for cancer theranostic applications.

## 1. Introduction

Cyanine dyes have shown great promise in biomedical applications [1,2]. Their versatile near-infrared (NIR) light responsive properties make them potential candidates for cancer treatment applications, such as photoacoustic imaging, NIR fluorescence imaging, photothermal therapy, and photodynamic therapy [3,4,5,6,7]. However, some of their inherent characteristics compromise their applications, such as undesirable stability, lack of tissue specificity, and rapid clearance from the body [8,9,10]. Many attempts have been performed to develop delivery systems to handle these limitations. Until now, different kinds of nanosystems have been developed to improve their biomedical applications, such as polymer nanoparticles (NPs) [11,12,13,14,15], inorganic nanomaterials [16,17,18], protein nanomaterials [19,20], carbon nanomaterials [21,22,23], and silica nanomaterials [24,25,26]. However, the loading ability of dendrimers for this kind of dyes has seldom been explored [27,28].

Dendrimers, especially poly(amidoamine) (PAMAM) dendrimers, have been demonstrated to be of great value in several kinds of biomedical application areas, such as tumor imaging, drug delivery, and gene delivery [29,30,31,32]. High generation PAMAM dendrimers (e.g., Generation 5, G5) possess well-defined composition and desirable monodispersity. Their nearly global architecture endows them with versatile ability for loading metal NPs and drugs [33,34,35]. Furthermore, they can be functionalized through the abundant surface amine groups to acquire improved biocompatibility and tissue specificity [36,37].

Among the normally used cyanine dyes, indocyanine green (ICG) and new indocyanine green (NICG, IR820) are drawing increasing attention [2,38]. Although owning similar chemical structures and optical/thermal generation properties, IR820 possesses a prominent absorption peak in the NIR region with improved stability [39]. Several strategies have been explored to improve their biomedical applications [38,40,41]. However, most of them lack tissue specificity, especially for tumors. Arginine-glycine-aspartic (RGD) peptide is a kind of popular targeting ligand, which shows specific binding to tumor endothelial cells through interaction with α_v_β_3_ integrin on the cell surface [42,43]. Through RGD modification, cargos could be delivered to cancer cells more effectively [44,45].

In this present work, multifunctional G5 PAMAM dendrimers were employed to be delivery systems for IR820 with enhanced stability and specificity. G5 PAMAM dendrimers with terminal amine groups (G5.NH_2_) were successively modified with RGD peptide through a poly(ethylene glycol) (PEG) linker and acetyl groups to improve their biocompatibility. Then the obtained G5.NHAc-PEG-RGD dendrimers were used to load IR820. The corresponding loading ability as well as the stability of the final products was investigated. Their cellular uptake behavior was studied by flow cytometry and confocal microscope.

## 2. Materials and Methods

### 2.1. Materials

Amine-terminated generation 5 poly(amidoamine) dendrimers were purchased from Dendritech (Midland, MI, USA). *N*-(3-Dimethylaminopropyl)-*N*′-ethylcarbodiimide hydrochloride (EDC), *N*-Hydroxysuccinimide (NHS), acetic anhydride (Ac_2_O), and triethylamine (Et_3_N) were obtained from J&K Chemical Reagent Co., Ltd. (Beijing, China). MAL-PEG-NHS (MW 2000, 90%) and mPEG-COOH (MW 2000, 95%) were obtained from Shanghai Yanyi Co., Ltd. (Shanghai, China). RGD peptide (RGD-SH) was provided by ChinaPeptides Co. Ltd. (Suzhou, China). IR820 (80%) was provided by Shanghai Titan Co., Ltd. (Shanghai, China). Dimethyl sulfoxide (DMSO) was provided by Greagent. All the chemicals were used without further purification. Dialysis bags with molecular weight cut-off (MWCO) of 1,000 and 14,000 Da were obtained from Shanghai Yuanye Biotechnology Corporation (Shanghai, China). U87MG cells (a human glioblastoma cell line) and L929 cells (a mouse fibroblast cell line) were obtained from the Institute of Biochemistry and Cell Biology, the Chinese Academy of Sciences (Shanghai, China). Minimum essential medium (MEM), trypsin containing EDTA, penicillin-streptomycin solution, and fetal bovine serum (FBS) were purchased from ThermoFisher Scientific (Waltham, MA, USA). Solutions of CCK-8 and Hoechst 33342 were obtained from Beyotime. De-ionized (DI) water (18.2 MΩ cm) from a water purification system (Synergy, Millipore, MA, USA) was used in all the preparation processes.

### 2.2. Preparation of Arginine-Glycine-Aspartic-Modified Dendrimers

G5.NH_2_ dendrimers were functionalized successively by RGD peptide, PEG chain, and acetyl groups according to the references, with some modifications [43,46]. Firstly, RGD-SH dissolved in DMSO was mixed with MAL-PEG-NHS of equivalent molar ratio. After reaction for 24 h, the products were purified by dialysis against DI water. Then, the obtained RGD-PEG-NHS was added into the G5.NH_2_ DMSO solution with the RGD/G5.NH_2_ molar ratio at 6/1. After 24 h reaction, mPEG-COOH that was pre-activated by 5-fold equivalent molar of EDC and NHS was added into the mixture, followed by stirring for 3 days. Finally, the residue surface amine groups were acetylated by using 6-fold equivalent molar of Et_3_N and 5-fold equivalent molar of Ac_2_O. The final products were dialyzed against DI water for 2 days and lyophilization to obtain G5.NHAc-PEG-RGD (Ac-PR) dendrimers.

For comparison, G5 dendrimers without RGD modification were also formed. The preparation protocol was similar to the aforementioned, only lacking feeding of RGD-PEG-NHS. The final obtained G5.NHAc-mPEG dendrimers were denoted as Ac-P.

### 2.3. Loading of New Indocyanine Green (IR820) Using the Obtained Dendrimers

Briefly, the aqueous solution of IR820 was fed into Ac-PR or Ac-P dendrimer solution at the IR820/dendrimer molar ratio at 30/1. After stirring in the dark for 4 h, the products were collected by centrifugation. The final obtained products were denoted as Ac-PR/IR820 and Ac-P/IR820, which were lyophilizated and stored at −20 °C for further use.

### 2.4. Characterization Techniques

Proton nuclear magnetic resonance (^1^H NMR) spectra of the dendrimers were measured via a Bruker AV 300 NMR (Karlsruhe, Germany) spectrometer in D_2_O solvent. Their UV-vis absorption spectra were obtained using a UV spectrophotometer (UV-1800, Shimadzu, Kyoto, Japan).

### 2.5. Stability Study

The optical property of the obtained Ac-PR/IR820 was selected as a parameter to evaluate their stability. Free IR820 was used as a control. Their UV-vis spectra were measured under different storage conditions. They were also dispersed in different kinds of solvent for observation.

The retaining efficiency of dendrimers for IR820 was investigated by a cumulative release experiment. Briefly, 2 mL of the Ac-PR/IR820 dendrimer solution (2 mg/mL) was enclosed in a semipermeable membrane (molecular weight cut-off = 14,000). The membrane was then immersed in a PBS solution (78 mL). The whole system was maintained at a constant temperature of 37 °C in a shaker. The buffer medium (0.1 mL) was withdrawn at predetermined time intervals. Their absorbance at 690 nm was measured using a microplate reader (SPARK 10M, Tecan, Männedorf, Switzerland) to evaluate the retain efficiency of dendrimers for IR820. 

### 2.6. Cell Culture

U87MG cells and L929 cells were regularly cultured and sub-cultured in minimum essential medium (MEM) supplemented with 10% fetal bovine serum (FBS) and 1% penicillin-streptomycin at 37 °C and 5% CO_2_ in a humidified incubator.

### 2.7. In Vitro Cytotoxicity Assay

The cytocompatibility of the obtained Ac-PR/IR820 dendrimers was quantified using a well-established CCK-8 colorimetric assay. Briefly, 1.5 × 10^4^ U87MG and L929 cells per well were co-incubated with Ac-PR/IR820 dendrimers of different concentrations. Cells treated with PBS were tested as control. After co-incubation, the medium was removed and each well was washed using PBS three times. After that, a standard CCK-8 assay was performed and the absorbance at 450 nm of the solution in each well was measured using a microplate reader (SPARK 10M, Tecan). The mean and standard deviation of measurements for triplicate wells were reported for each sample.

### 2.8. Cellular Uptake and Intracellular Localization Study

The cellular uptake behaviors of Ac-PR/IR820 dendrimers were investigated using flow cytometry analysis. Ac-P/IR820 dendrimers and PBS were also tested as a contrast and a blank control, respectively. Briefly, 1.0 × 10^5^ cells per well were seeded into a 24-well plate. After one day, the medium was replaced with fresh medium containing dendrimers at different IR820 concentrations. After co-incubation for 3 h or 6 h, the medium was aspirated and each well was washed thrice with PBS. The cells were trypsinized, resuspended in PBS, and analyzed via flow cytometry (NovoCyte, ACEA, San Diego, CA, USA). For each sample, 1 × 10^4^ cell events were measured and analyzed. For the receptor blocking experiment, U87MG cells were pre-incubated with free RGD (2 μM) for 3 h, followed by a protocol similar to the above-mentioned protocol.

A confocal laser-scanning microscopy was further employed to study their cellular uptake behaviors, as well as their intracellular localization. In this experiment, 2 × 10^5^ U87MG cells were seeded in 12-well cell-containing glass slides. After one day, the medium was replaced with fresh medium containing dendrimers at an IR820 final concentration of 2.5 μM. After co-incubation for 6 h, the medium was aspirated and each well was washed thrice with PBS. Thereafter, the cells were incubated with Hoechst 33,342 to stain the nucleus in accordance with the manufacturer’s instructions. Multiple laser channels (excitation wavelengths = 455 nm and 773 nm) were utilized to excite Hoechst 33,342 and IR820, respectively. The fluorescence emissions through the corresponding channels were recorded using confocal laser-scanning microscopy (LSM 780, Carl Zeiss, Oberkochen, Germany).

## 3. Results and Discussion

### 3.1. Preparation and Characterization of Multifunctional IR820-Loaded Dendrimers

**Figure 1:**
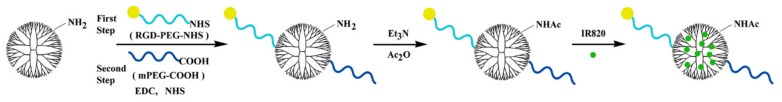
Schematic illustration of the synthesis of Ac-PR/IR820 dendrimers.

**Figure 2:**
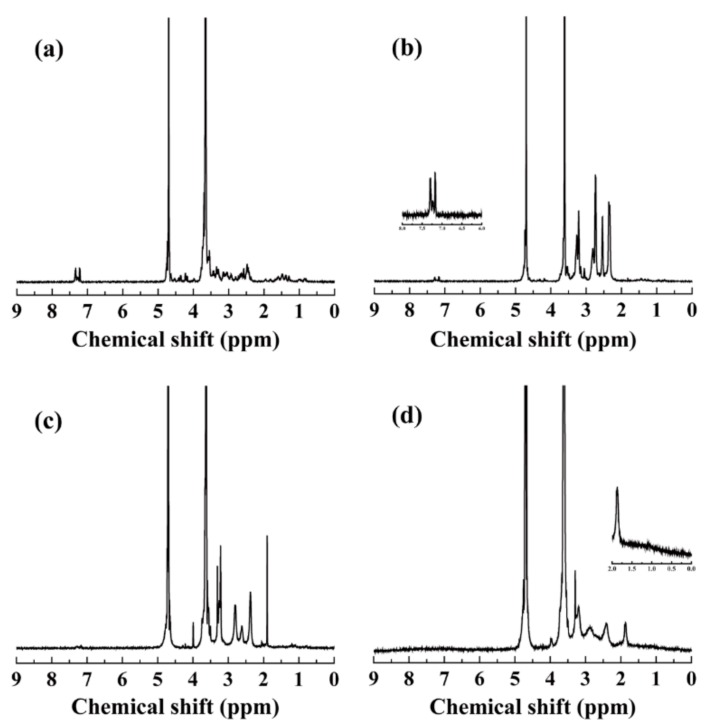
(**a**) ^1^H NMR spectra of RGD-PEG-NHS; (**b**) ^1^H NMR spectra of G5.NH_2_-PEG-RGD dendrimers; (**c**) ^1^H NMR spectra of Ac-PR dendrimers; and (**d**) ^1^H NMR spectra of Ac-PR/IR820 dendrimers.

The preparation process of the multifunctional IR820-loaded dendrimers was illustrated in Figure 1. Amine-terminated G5 PAMAM dendrimers were chosen as multifunctional carriers for IR820 because of their abundant inner cavities and surface modifiable groups. Firstly, RGD-SH was pre-linked to MAL-PEG-NHS through the reaction between –SH and –MAL. The successful linkage was proved by ^1^H NMR spectrum (Figure 2a). The peak around 3.6 ppm and 7.4 ppm could be assigned to PEG chain and RGD moiety, respectively [46]. Then the formed RGD-PEG-NHS and pre-activated mPEG-COOH were modified onto the G5.NH_2_ dendrimer surface (Figure 2b). Through the calculation of the integration of the corresponding peaks, the number of PEG and RGD attached onto each dendrimer was estimated to be 34.3 and 3.2, respectively. Then, the residue amine groups were transformed to acetyl groups, which was confirmed by the newly appeared peak around 1.8 ppm (Figure 2c) [33]. The number of acetyl groups per dendrimer was calculated to be 57.3, according to their corresponding integrations of the peaks. The formed Ac-PR dendrimers were then employed to load IR820. The signals from 1.5 to 0.5 ppm were attributed to the overlap of RGD and IR820. By deducting the integration of RGD moieties, the number of IR820 was estimated to be 6.7 per dendrimer (Figure 2d). Compared with most of the delivery systems for IR820 [2,3], dendrimer-based systems could make quantification analysis of IR820 on each NP possible, which will benefit further quantitative research in vitro and in vivo.

**Figure 3:**
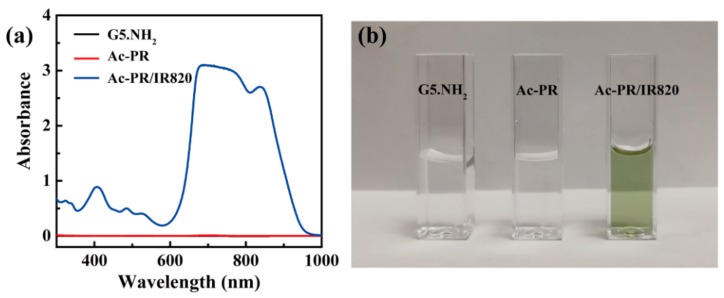
UV-vis spectra of G5.NH_2_, Ac-PR, and Ac-PR/IR820 dendrimers (**a**) and their corresponding photos (**b**).

UV-vis spectra were used to investigate the optical properties of the dendrimers (Figure 3a). G5.NH_2_ dendrimers showed negligible absorbance in the wavelength range of 1000 to 300 nm. After RGD and PEG modification, no obvious changes were observed. After IR820 loading, a broad peak appeared in the wavelength range of 1000 to 600 nm. It can also be seen from the photos of their aqueous solution (Figure 3b). Before IR820 loading, the solutions of the dendrimers were colorless. A bright green color can be observed after IR820 loading. The hydrodynamic size and surface potential of the formed Ac-PR/IR820 dendrimers were measured to be 337.1 ± 41.4 nm, with a polydispersity of 0.893 ± 0.096 and 10.0 ± 0.6 mV.

In addition, dendrimers without RGD modification were also prepared as control dendrimers using a similar protocol. G5.NH_2_ dendrimers were successively modified with mPEG-COOH and acetyl groups, followed by IR820 loading. The formed dendrimers were also characterized by ^1^H NMR spectra (Figure S1). The number of loaded IR820 per dendrimer was estimated to be 6.4, which was similar to that of Ac-PR dendrimers. Their optical properties were also studied using UV-vis spectra, which were similar to that of Ac-PR dendrimers (Figure S2a). However, the solution color of Ac-P/IR820 dendrimers was purple, which was quite different from that of Ac-P/IR820 dendrimers (Figure S2b). This may be caused by the different loading interaction between dendrimers and IR820, with or without the RGD moiety. 

### 3.2. Stability Study

**Figure 4:**
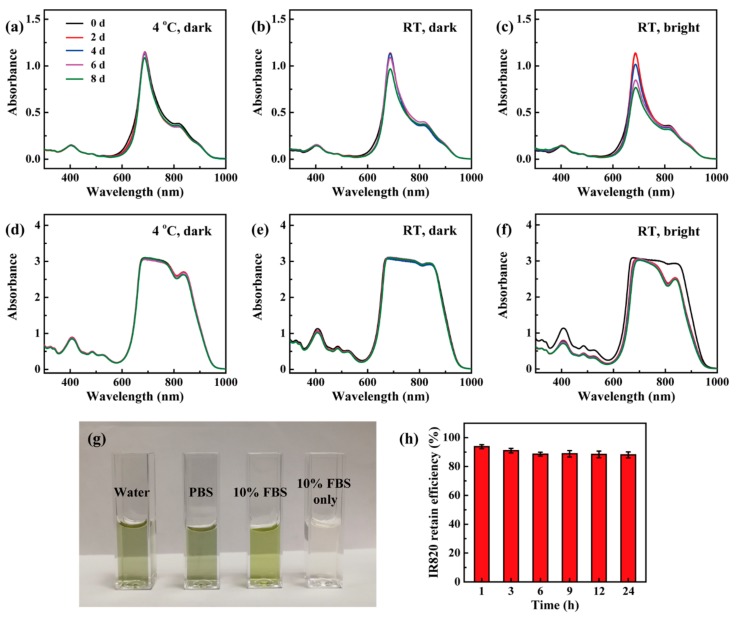
UV-vis spectra of free IR820 (**a**–**c**) and Ac-PR/IR820 (**d**–**f**) dendrimers when stored at distinct conditions; (**g**) the photo of Ac-PR/IR820 dendrimers dispersed in kinds of solvents; (**h**) the retain efficiency of Ac-PR dendrimers for IR820.

The stability of the formed Ac-PR/IR820 dendrimers was characterized and compared with free IR820. When stored at 4 °C in dark conditions, IR820 displayed similar optical spectra within eight days, displaying desirable stability (Figure 4a). For room temperature (RT, approximate 25 °C) and dark storage conditions, the UV-vis spectra kept similar with six days, which showed a 15% decrease at the absorbance peak (Figure 4b). Its absorbance spectra showed continuous decrease at the absorbance peak when stored at RT and bright conditions (Figure 4c). When loaded by dendrimers, their UV-vis spectra were similar when stored in dark conditions at 4 °C and RT (Figure 4d,e). When stored at RT and dark conditions, their UV-vis spectra also kept similar without continuous decrease, only with some change in the wavelength around 830 nm (Figure 4f). It could be noticed that the UV-vis curves around 830 nm were different under kinds of storage conditions. This may reflect that the interactions between IR820 and dendrimers are viable under different temperature and brightness conditions. In addition, the formed Ac-PR/IR820 dendrimers dispersed well in different kinds of solvents (Figure 4g). Importantly, Ac-PR dendrimers could retain the loaded IR820 steadily, showing a retaining efficiency as high as 88.0% after incubated in PBS for 24 h (Figure 4h). All these data proved that the stability of IR820 was improved through dendrimer loading, and the formed dendrimer/IR820 complex was stable under tested conditions. 

### 3.3. In Vitro Cytocompatibility Assay

**Figure 5:**
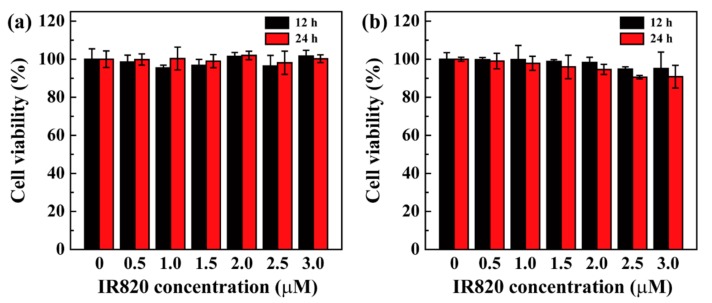
Relative viability of U87MG cells (**a**) and L929 cells (**b**) co-incubated with Ac-PR/IR820 dendrimers for 12 h and 24 h, measured by CCK-8 assay.

The cytocompatibility of nanomaterials is an important parameter to evaluate their potential biomedical applications. Herein, a well-established CCK-8 method was employed to evaluate the cytotoxicity of the formed Ac-PR/IR820 dendrimers using U87MG cells and L929 cells as a model. After co-incubation with Ac-PR/IR820 dendrimers at IR820 concentrations from 0.5 to 3.0 μM, all measured cell viabilities were similar to those of PBS-treated cells (Figure 5). No obvious decrease in cell viability was observed at 12 and 24 h co-incubation times. Based on these data, it can be assumed that the formed Ac-PR/IR820 dendrimers have desirable cytocompatibility.

### 3.4. Cellular Uptake and Intracellular Localization Study

**Figure 6:**
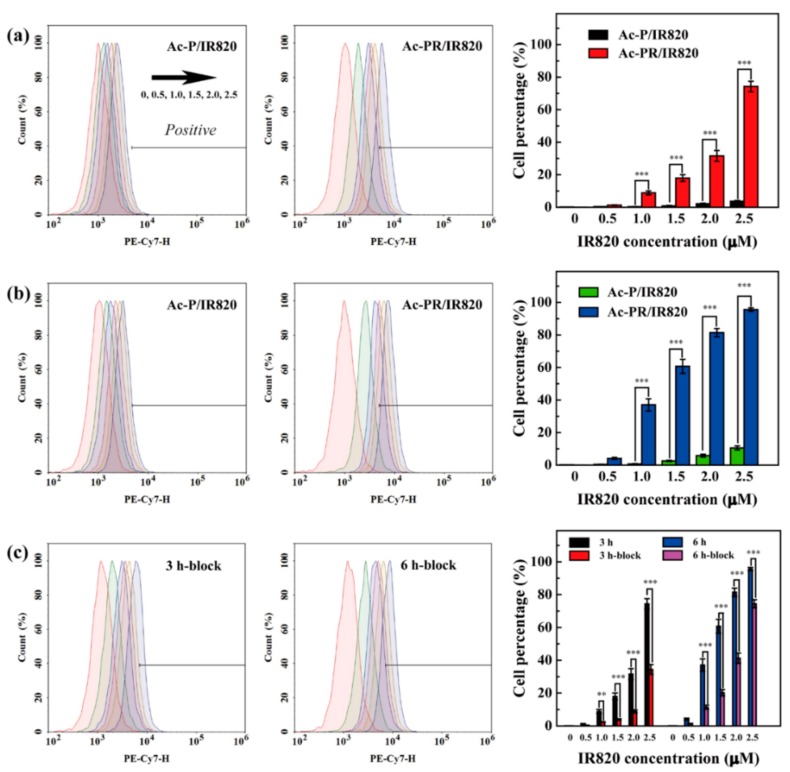
Flow cytometry analysis of U87MG cells after co-incubation with Ac-P/IR820 and Ac-PR/IR820 dendrimers for 3 h (**a**) and 6 h (**b**). U87MG cells pre-incubated with free RGD (2 μM) for 3 h and then co-incubated with Ac-PR/IR820 dendrimers for 3 h and 6 h were also tested for comparison (**c**) (* for *p* < 0.05, ** for *p* < 0.01, and *** for *p* < 0.001, respectively).

Flow cytometry assay was performed to assess the cellular uptake behaviors of IR820 loaded by dendrimers. Using U87MG cells as a model cell line, their cellular uptake behaviors were measured and analyzed. After a 3-h co-incubation (Figure 6a), the percentage of IR820 fluorescence-positive cells was found to have increased with the NP concentrations for both dendrimers. The cell percentages increased for all conditions after 6 h of co-incubation (Figure 6b). The cells co-incubated with Ac-PR/IR820 dendrimers displayed a fluorescence-positive percentage of 96.55%, indicating the effective uptake of Ac-PR/IR820 dendrimers by the U87MG cells. For both conditions (concentration range of 1.0 to 2.5 μM at 3 h and 6 h), IR820 loaded by Ac-PR dendrimers showed much higher cell uptake efficiency, with significance difference to that loaded by Ac-P dendrimers. This indicated that RGD modification could endow cell specificity for the dendrimers, leading to enhanced cellular uptake behaviors.

This specificity was further proved by a blocking experiment. When the cells were pre-incubated with free RGD, their surface α_v_β_3_ integrin receptors were blocked. After co-incubation with Ac-PR/IR820 dendrimers for 3 h and 6 h, their cellular uptake percentages decreased significantly, when compared to the cells without blocking (Figure 6c). When incubated with L929 cells (lack of α_v_β_3_ integrin receptors), similar cellular uptake behaviors were observed for Ac-P/IR820 and Ac-PR/IR820 dendrimers (Figure S3), also indicating the RGD-mediated cellular uptake. 

**Figure 7:**
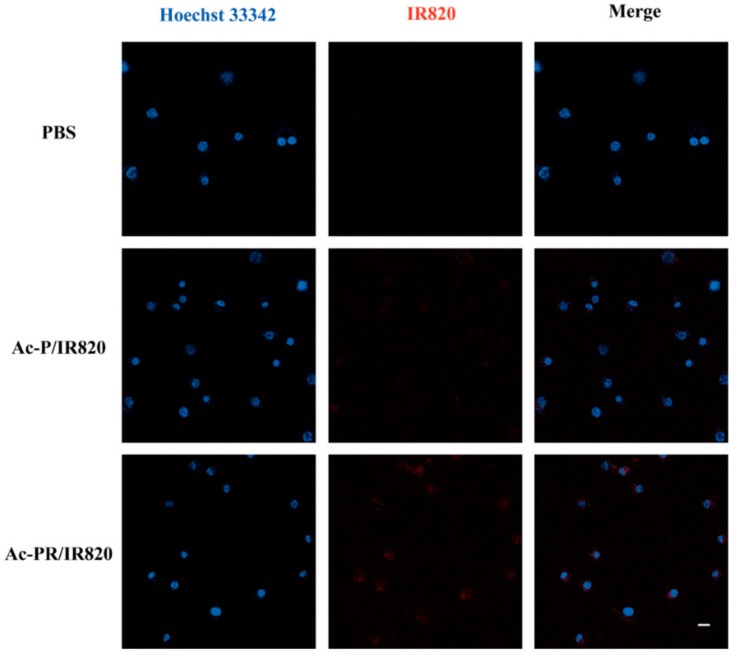
Confocal fluorescence images of U87MG cells after 6 h co-incubation with IR820-loaded dendrimers. Cells treated with PBS were tested as control. The fluorescence of Hoechst 33342 and IR820 were pseudo-labeled with blue and red, respectively. Scale bars: 20 μm.

The intracellular localization of the internalized dendrimers was observed using laser scanning confocal microscopy (Figure 7). After co-incubation with dendrimers at IR820 concentration of 2.5 μM for 6 h, Ac-PR/IR820 dendrimers displayed higher fluorescence than Ac-P/IR820 dendrimers, indicating an enhanced cellular uptake. It could be seen from the images that most internalized dendrimers were located in cytoplasm, surrounding the cell nuclei.

## 4. Conclusions

In summary, amine-terminated G5 PAMAM dendrimers were employed to construct a targeted delivery system for IR820. G5 dendrimers were successfully modified with RGD peptides, PEG chains, and acetyl groups. The formed Ac-PR dendrimers can load IR820 effectively. The formed Ac-PR/IR820 dendrimers were stable under different kinds of storage conditions, showing improved stability compared with free IR820. The cytocompatibility of the formed Ac-PR/IR820 dendrimers were desirable under the studied conditions. Compared with non-targeted dendrimers, the cellular uptake behaviors were demonstrated to be enhanced by RGD modification, showing concentration-, co-incubation time-, and α_v_β_3_ integrin receptor-dependent properties. The internalized dendrimers mostly displayed a cytoplasm-location. The findings from this work demonstrated the versatile loading and delivery capacity of dendrimer for NIR dyes, which were promising in potential cancer theranostic applications.

## Supplementary Materials

The following are available online at http://www.mdpi.com/1999-4923/10/3/77/s1, Figure S1: ^1^H NMR spectra of G5.NH_2_-mPEG (a); Ac-P (b); and Ac-P/IR820 (c); Figure S2: UV-vis spectra G5.NH_2_, Ac-P, and Ac-P/IR820 dendrimers (a) and their corresponding photos (b).

## References

[1] Cai L., Sheng Z., Hu D., Xue M., He M., Gong P. (2013). Indocyanine green nanoparticles for theranostic applications. Nano-Micro Lett..

[2] Wang H., Li X., Tse B.W., Yang H., Thorling C.A., Liu Y., Touraud M., Chouane J.B., Liu X., Roberts M.S. (2018). Indocyanine green-incorporating nanoparticles for cancer theranostics. Theranostics.

[3] Li Y., Liu G., Ma J., Lin J., Lin H., Su G., Chen D., Ye S., Chen X., Zhu X. (2017). Chemotherapeutic drug-photothermal agent co-self-assembling nanoparticles for near-infrared fluorescence and photoacoustic dual-modal imaging-guided chemo-photothermal synergistic therapy. J. Control. Release.

[4] Chen Z., Zhao P., Luo Z., Zheng M., Tian H., Gong P., Gao G., Pan H., Liu L., Ma A. (2016). Cancer cell membrane–biomimetic nanoparticles for homologous-targeting dual-modal imaging and photothermal therapy. ACS Nano.

[5] Wang G., Zhang F., Tian R., Zhang L., Fu G., Yang L., Zhu L. (2016). Nanotubes-embedded indocyanine green-hyaluronic acid nanoparticles for photoacoustic-imaging-guided phototherapy. ACS Appl. Mater. Interfaces.

[6] Liu Y., Zhi X., Yang M., Zhang J., Lin L., Zhao X., Hou W., Zhang C., Zhang Q., Pan F. (2017). Tumor-triggered drug release from calcium carbonate-encapsulated gold nanostars for near-infrared photodynamic/photothermal combination antitumor therapy. Theranostics.

[7] Fang S., Lin J., Li C., Huang P., Hou W., Zhang C., Liu J., Huang S., Luo Y., Fan W. (2017). Dual-stimuli responsive nanotheranostics for multimodal imaging guided trimodal synergistic therapy. Small.

[8] Zhou H., Hou X., Liu Y., Zhao T., Shang Q., Tang J., Liu J., Wang Y., Wu Q., Luo Z. (2016). Superstable magnetic nanoparticles in conjugation with near-infrared dye as a multimodal theranostic platform. ACS Appl. Mater. Interfaces.

[9] Gao F., Bai L., Feng X., Tham H.P., Zhang R., Zhang Y., Liu S., Zhao L., Zheng Y., Zhao Y. (2016). Remarkable in vivo nonlinear photoacoustic imaging based on near-infrared organic dyes. Small.

[10] Dong Z., Gong H., Gao M., Zhu W., Sun X., Feng L., Fu T., Li Y., Liu Z. (2016). Polydopamine nanoparticles as a versatile molecular loading platform to enable imaging-guided cancer combination therapy. Theranostics.

[11] Hu D., Liu C., Song L., Cui H., Gao G., Liu P., Sheng Z., Cai L. (2016). Indocyanine green-loaded polydopamine-iron ions coordination nanoparticles for photoacoustic/magnetic resonance dual-modal imaging-guided cancer photothermal therapy. Nanoscale.

[12] Li N., Li T., Hu C., Lei X., Zuo Y., Han H. (2016). Targeted near-infrared fluorescent turn-on nanoprobe for activatable imaging and effective phototherapy of cancer cells. ACS Appl. Mater. Interfaces.

[13] Hu D., Zhang J., Gao G., Sheng Z., Cui H., Cai L. (2016). Indocyanine green-loaded polydopamine-reduced graphene oxide nanocomposites with amplifying photoacoustic and photothermal effects for cancer theranostics. Theranostics.

[14] Liao J., Wei X., Ran B., Peng J., Qu Y., Qian Z. (2017). Polymer hybrid magnetic nanocapsules encapsulating IR820 and PTX for external magnetic field-guided tumor targeting and multifunctional theranostics. Nanoscale.

[15] Mehnath S., Rajan M., Sathishkumar G., Praphakar R.A., Jeyaraj M. (2017). Thermoresponsive and pH triggered drug release of cholate functionalized poly(organophosphazene)—Polylactic acid copolymeric nanostructure integrated with ICG. Polymer.

[16] Liu C., Chen J., Zhu Y., Gong X., Zheng R., Chen N., Chen D., Yan H., Zhang P., Zheng H. (2018). Highly sensitive MoS2-indocyanine green hybrid for photoacoustic imaging of orthotopic brain glioma at deep site. Nano-Micro Lett..

[17] Zhang H., Zhang X., Zhu X., Chen J., Chen Q., Zhang H., Hou L., Zhang Z. (2018). NIR light-induced tumor phototherapy using photo-stable ICG delivery system based on inorganic hybrid. Nanomed.-Nanotechnol..

[18] Park H.S., Kim J., Cho M.Y., Lee H., Nam S.H., Suh Y.D., Hong K.S. (2017). Convenient and effective ICGylation of magnetic nanoparticles for biomedical applications. Sci. Rep..

[19] You Q., Sun Q., Yu M., Wang J.P., Wang S.Y., Liu L., Cheng Y., Wang Y.D., Song Y.L., Tan F.P. (2017). BSA-bioinspired gadolinium hybrid-functionalized hollow gold nanoshells for NIRF/PA/CT/MR quadmodal diagnostic imaging guided photothermal/photodynamic cancer therapy. ACS Appl. Mater. Interfaces.

[20] Sahu A., Lee J.H., Lee H.G., Jeong Y.Y., Tae G. (2016). Prussian blue/serum albumin/indocyanine green as a multifunctional nanotheranostic agent for bimodal imaging guided laser mediated combinatorial phototherapy. J. Control. Release.

[21] Miao W., Shim G., Kim G., Lee S., Lee H.J., Kim Y.B., Byun Y., Oh Y.K. (2015). Image-guided synergistic photothermal therapy using photoresponsive imaging agent-loaded graphene-based nanosheets. J. Control. Release.

[22] Wang Y.-W., Fu Y.-Y., Peng Q., Guo S.-S., Liu G., Li J., Yang H.-H., Chen G.-N. (2013). Dye-enhanced graphene oxide for photothermal therapy and photoacoustic imaging. J. Mater. Chem. B.

[23] Zanganeh S., Li H., Kumavor P.D., Alqasemi U., Aguirre A., Mohammad I., Stanford C., Smith M.B., Zhu Q. (2013). Photoacoustic imaging enhanced by indocyanine green-conjugated single-wall carbon nanotubes. J. Biomed. Opt..

[24] Xia B., Zhang Q., Shi J., Li J., Chen Z., Wang B. (2018). Co-loading of photothermal agents and anticancer drugs into porous silicon nanoparticles with enhanced chemo-photothermal therapeutic efficacy to kill multidrug-resistant cancer cells. Colloid Surf. B.

[25] Hong S.H., Kim H., Choi Y. (2017). Indocyanine green-loaded hollow mesoporous silica nanoparticles as an activatable theranostic agent. Nanotechnology.

[26] Xia B., Wang B., Chen Z., Zhang Q., Shi J. (2016). Near-infrared light-triggered intracellular delivery of anticancer drugs using porous silicon nanoparticles conjugated with IR820 dyes. Adv. Mater. Interfaces.

[27] Zan M., Li J., Huang M., Lin S., Luo D., Luo S., Ge Z. (2015). Near-infrared light-triggered drug release nanogels for combined photothermal-chemotherapy of cancer. Biomater. Sci..

[28] Fang M., Zhang J., Wu Q., Xu T., Cheng Y. (2012). Host-guest chemistry of dendrimer-drug complexes: 7. Formation of stable inclusions between acetylated dendrimers and drugs bearing multiple charges. J. Phys. Chem. B.

[29] Kesharwani P., Gothwal A., Iyer A.K., Jain K., Chourasia M.K., Gupta U. (2017). Dendrimer nanohybrid carrier systems: An expanding horizon for targeted drug and gene delivery. Drug Discov. Today.

[30] Mignani S., Rodrigues J., Tomas H., Zablocka M., Shi X., Caminade A.-M., Majoral J.-P. (2018). Dendrimers in combination with natural products and analogues as anti-cancer agents. Chem. Soc. Rev..

[31] Qiao Z., Shi X. (2015). Dendrimer-based molecular imaging contrast agents. Prog. Polym. Sci..

[32] Mignani S., Bryszewska M., Zablocka M., Klajnert-Maculewicz B., Cladera J., Shcharbin D., Majoral J.-P. (2017). Can dendrimer based nanoparticles fight neurodegenerative diseases? Current situation versus other established approaches. Prog. Polym. Sci..

[33] Peng C., Zheng L., Chen Q., Shen M., Guo R., Wang H., Cao X., Zhang G., Shi X. (2012). PEGylated dendrimer-entrapped gold nanoparticles for in vivo blood pool and tumor imaging by computed tomography. Biomaterials.

[34] Xiong Z., Wang Y., Zhu J., Li X., He Y., Qu J., Shen M., Xia J., Shi X. (2017). Dendrimers meet zwitterions: Development of a unique antifouling nanoplatform for enhanced blood pool, lymph node and tumor CT imaging. Nanoscale.

[35] Zhu J., Zheng L., Wen S., Tang Y., Shen M., Zhang G., Shi X. (2014). Targeted cancer theranostics using alpha-tocopheryl succinate-conjugated multifunctional dendrimer-entrapped gold nanoparticles. Biomaterials.

[36] Lin L., Fan Y., Gao F., Jin L., Li D., Sun W., Li F., Qin P., Shi Q., Shi X. (2018). UTMD-promoted co-delivery of gemcitabine and miR-21 inhibitor by dendrimer-entrapped gold nanoparticles for pancreatic cancer therapy. Theranostics.

[37] Ma W., Fu F., Zhu J., Huang R., Zhu Y., Liu Z., Wang J., Conti P.S., Shi X., Chen K. (2018). Cu-64-Labeled multifunctional dendrimers for targeted tumor PET imaging. Nanoscale.

[38] Yang W., Noh J., Park H., Gwon S., Singh B., Song C., Lee D. (2018). Near infrared dye-conjugated oxidative stress amplifying polymer micelles for dual imaging and synergistic anticancer phototherapy. Biomaterials.

[39] Fernandez-Fernandez A., Manchanda R., Lei T., Carvajal D.A., Tang Y., Kazmi S.Z.R., McGoron A.J. (2012). Comparative study of the optical and heat generation properties of IR820 and indocyanine green. Mol. Imaging.

[40] Li T., Shen X., Xie X., Chen Z., Li S., Qin X., Yang H., Wu C., Liu Y. (2018). Irinotecan/IR-820 coloaded nanocomposite as a cooperative nanoplatform for combinational therapy of tumor. Nanomedicine.

[41] Zhao Q., Wang X., Yan Y., Wang D., Zhang Y., Jiang T., Wang S. (2017). The advantage of hollow mesoporous carbon as a near-infrared absorbing drug carrier in chemo-photothermal therapy compared with IR-820. Eur. J. Pharm. Sci..

[42] Yan F., Wu H., Liu H., Deng Z., Liu H., Duan W., Liu X., Zheng H. (2016). Molecular imaging-guided photothermal/photodynamic therapy against tumor by iRGD-modified indocyanine green nanoparticles. J. Control. Release.

[43] Xu X., Zhao L., Li X., Wang P., Zhao J., Shi X., Shen M. (2017). Targeted tumor SPECT/CT dual mode imaging using multifunctional RGD-modified low generation dendrimer-entrapped gold nanoparticles. Biomater. Sci..

[44] Li Y., Jiang C., Zhang D., Wang Y., Ren X., Ai K., Chen X., Lu L. (2017). Targeted polydopamine nanoparticles enable photoacoustic imaging guided chemo-photothermal synergistic therapy of tumor. Acta Biomater..

[45] Ding X., Hao X., Fu D., Zhang M., Lan T., Li C., Huang R., Zhang Z., Li Y., Wang Q. (2017). Gram-scale synthesis of nanotherapeutic agents for CT/T1-weighted MRI bimodal imaging guided photothermal therapy. Nano Res..

[46] He X., Alves C.S., Oliveira N., Rodrigues J., Zhu J., Bányai I., Tomás H., Shi X. (2015). RGD peptide-modified multifunctional dendrimer platform for drug encapsulation and targeted inhibition of cancer cells. Colloid. Surface B.

