# Supplementary material for: Loading IR820 Using Multifunctional Dendrimers with Enhanced Stability and Specificity
Source: Pharmaceutics. 2018 Jun 28;10(3):77. doi: 10.3390/pharmaceutics10030077 (PMC6161036; doi:10.3390/pharmaceutics10030077)
Supplement: Supplementary file 1 [file pharmaceutics-10-00077-s001.pdf]

pharmaceutics

Supplementary Materials for Loading IR820 using Multifunctional Dendrimers with Enhanced Stability and Specificity

Hui Liu * and Jingjing Wang

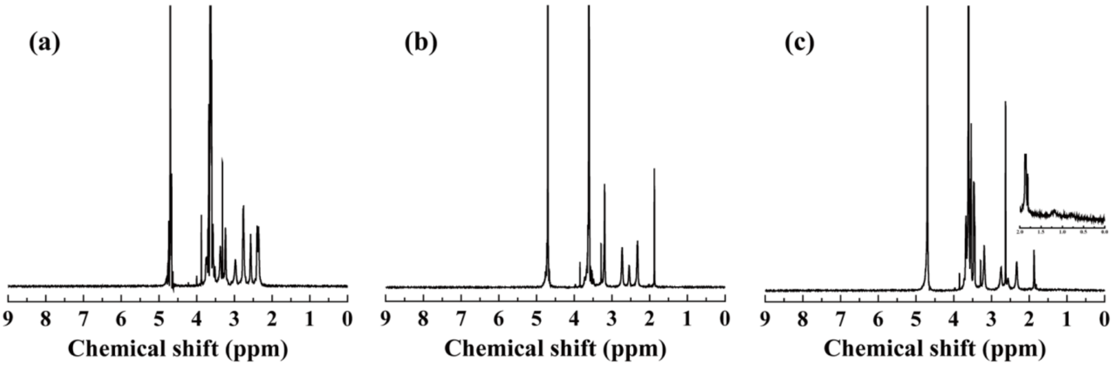

Figure S1. ^{1}HNMR spectra of G5.NH_2-mPEG (**a**), Ac-P (**b**), and Ac-P/IR820 (**c**).

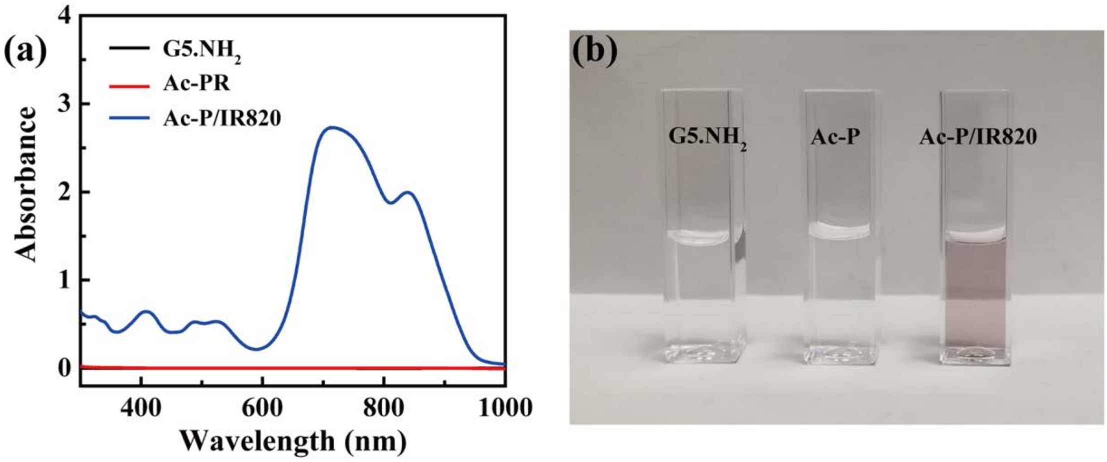

Figure S2. UV-vis spectra G5.NH_2, Ac-P, and Ac-P/IR820 dendrimers (**a**) and their corresponding photos (**b**).

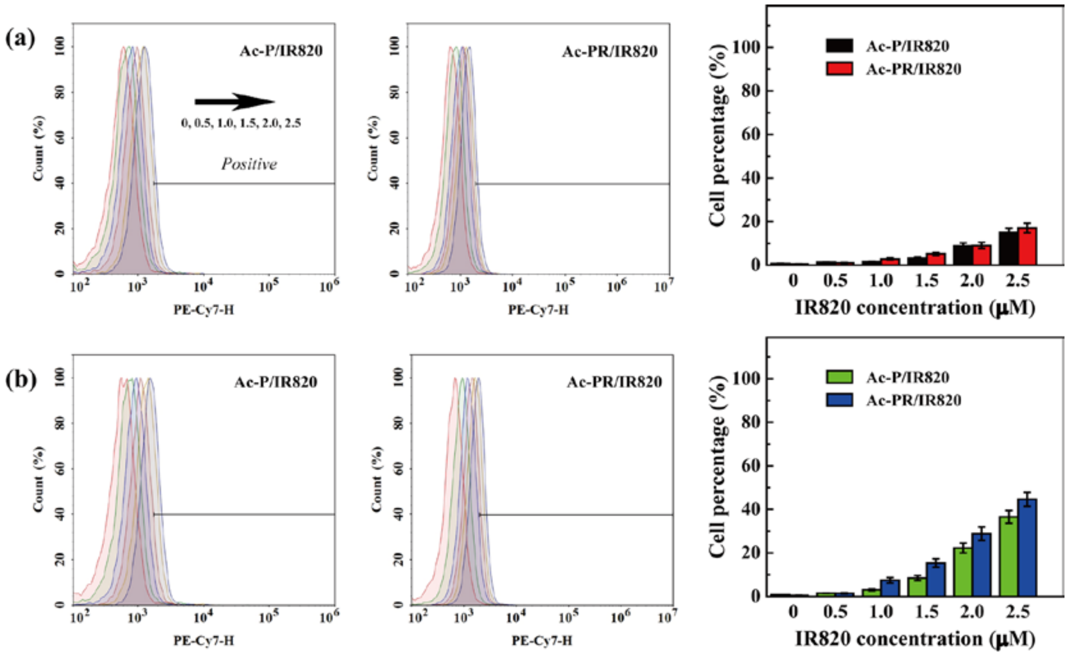

Figure S3. Flow cytometry analysis of L929 cells after co-incubation with Ac-P/IR820 and Ac-PR/IR820 dendrimers for 3 h (**a**) and 6 h (**b**).
